# Magnetic resonance imaging evaluation after implantation of a titanium cervical disc prosthesis: a comparison of 1.5 and 3 Tesla magnet strength

**DOI:** 10.1007/s00586-013-2994-z

**Published:** 2013-09-06

**Authors:** Jarle Sundseth, Eva A. Jacobsen, Frode Kolstad, Oystein P. Nygaard, John A. Zwart, Per K. Hol

**Affiliations:** 1 Department of Neurosurgery, Oslo University Hospital Rikshospitalet, Oslo, Norway; 2 University of Oslo, Oslo, Norway; 3 Department of Neuroradiology, Oslo University Hospital Rikshospitalet, Oslo, Norway; 4 Department of Neurosurgery, University Hospital of Trondheim, St Olavs Hospital HF, Trondheim, Norway; 5 University of Trondheim, Trondheim, Norway; 6 Department of Neurology and FORMI, Oslo University Hospital Ullevaal, Oslo, Norway; 7 The Intervention Center, Oslo University Hospital Rikshospitalet, Oslo, Norway; 8 Department of Neurosurgery, Oslo University Hospital Rikshospitalet, Nydalen, Postboks 4950, 0424 Oslo, Norway

**Keywords:** Magnetic resonance imaging, Artifact, Cervical disc prostheses, Titanium

## Abstract

**Purpose:**

Cervical disc prostheses induce significant amount of artifact in magnetic resonance imaging which may complicate radiologic follow-up after surgery. The purpose of this study was to investigate as to what extent the artifact, induced by the frequently used Discover^®^ cervical disc prosthesis, impedes interpretation of the MR images at operated and adjacent levels in 1.5 and 3 Tesla MR.

**Methods:**

Ten subsequent patients were investigated in both 1.5 and 3 Tesla MR with standard image sequences one year following anterior cervical discectomy with arthroplasty.

**Outcome measures:**

Two neuroradiologists evaluated the images by consensus. Emphasis was made on signal changes in medulla at all levels and visualization of root canals at operated and adjacent levels. A “blur artifact ratio” was calculated and defined as the height of the artifact on T1 sagittal images related to the operated level.

**Results:**

The artifacts induced in 1.5 and 3 Tesla MR were of entirely different character and evaluation of the spinal cord at operated level was impossible in both magnets. Artifacts also made the root canals difficult to assess at operated level and more pronounced in the 3 Tesla MR. At the adjacent levels however, the spinal cord and root canals were completely visualized in all patients. The “blur artifact” induced at operated level was also more pronounced in the 3 Tesla MR.

**Conclusions:**

The artifact induced by the Discover^®^ titanium disc prosthesis in both 1.5 and 3 Tesla MR, makes interpretation of the spinal cord impossible and visualization of the root canals difficult at operated level. Adjusting the MR sequences to produce the least amount of artifact is important.

## Introduction

The Anterior Cervical Discectomy and Fusion (ACDF) has become a standard surgical procedure for treating degenerative cervical disc disease causing radiculopathy or myelopathy [1–4]. In the late 1990s, Anterior Cervical Discectomy with Arthroplasty (ACDA) was introduced as an alternative to fusion, based on the notion that preserving motion reduces the risk of adjacent level degeneration [5, 6]. Arthroplasty is found in some of these studies to be superior to ACDF regarding clinical outcome as well as maintaining motion and preventing adjacent level disease [7, 8]. On the other hand, meta-analyses of existing prospective, randomized, controlled trials in 2010 and 2012 comparing ACDA with ACDF, conclude that clinical benefit for the cervical disc prosthesis is not proved [9, 10]. Even though the clinical outcome using either arthroplasty or fusion is well documented, there are some patients who will experience persistent or increasing symptoms over time. In such cases, it will be necessary to evaluate both the spinal cord and root canals at operated and adjacent levels. Magnetic resonance imaging (MRI) is considered the ideal screening method for investigation of patients with cervical myelopathy or radiculopathy, and is preferred to computed tomography and myelography due to its high soft tissue contrast discrimination and noninvasiveness [11–13]. Metallic implants are, however, known to induce artifacts in MRI which may impede interpretation of the images [14]. The titanium produced cervical disc prostheses Discover^®^ (DePuy Spine, Inc. 325 Paramount Drive Raynham, MA 02767-0350 USA) is stated to be MR compatible, in the sense that patients with this prosthesis can undergo MR examination. However, artifacts will appear in the images [15] and are assumed to be different in examinations performed in 3 Tesla (T) scanners compared to 1.5 T, as shown concerning the artifacts related to aneurysm clips and shunt valves [16].

We conducted this study to determine the extent of artifact induced by this disc prosthesis and how it limits interpretation of the MRI in a 1.5 T magnet compared to a 3 T magnet.

## Materials and methods

Ten subsequent patients, one year following ACDA, were investigated in 3 T (Achieva, Philips Healthcare, Best, The Netherlands) and 1.5 T (Siemens Symphony, Erlangen, Germany) MRs. All patients were participants in a prospective, randomized controlled clinical multicenter study on 1-level ACDA versus 1-level ACDF [17].

The surgical procedure was performed with the patient in the supine position and under general anesthesia. A standard anterior approach was used to reach the cervical disc. The disc was then removed and the nerve root decompressed. After decompression of the nerve root, the patient was randomized to either implantation of the Discover^®^ cervical disc prostheses or the Cervios PolyEtherEtherKetone (PEEK) cage (SYNTHES^®^ GmbH Eimattstrasse 3 CH-4436 Oberdorf).

Inclusion criteria for the randomized controlled multicenter study were clinical C6 or C7 root radiculopathy with corresponding radiological findings, Neck Disability Index (NDI) equal to or more than 30 points, no effect of conservative treatment and no signs of improvement during the last 6 weeks prior to surgery. From March 2009 to January 2013, 143 patients were included in the study at five hospitals in Norway. Fifty-four percent were operated at level C5/C6 and the remaining at level C6/C7. According to protocol all patients underwent MRI at 3, 12 and 24 months after surgery.

The first 10 participating patients, who were randomized to ACDA at the Oslo University Hospital (OUS) Rikshospitalet, were at 12-month follow-up assessed with both 1.5 and 3 T MRI.

**Fig. 1:**
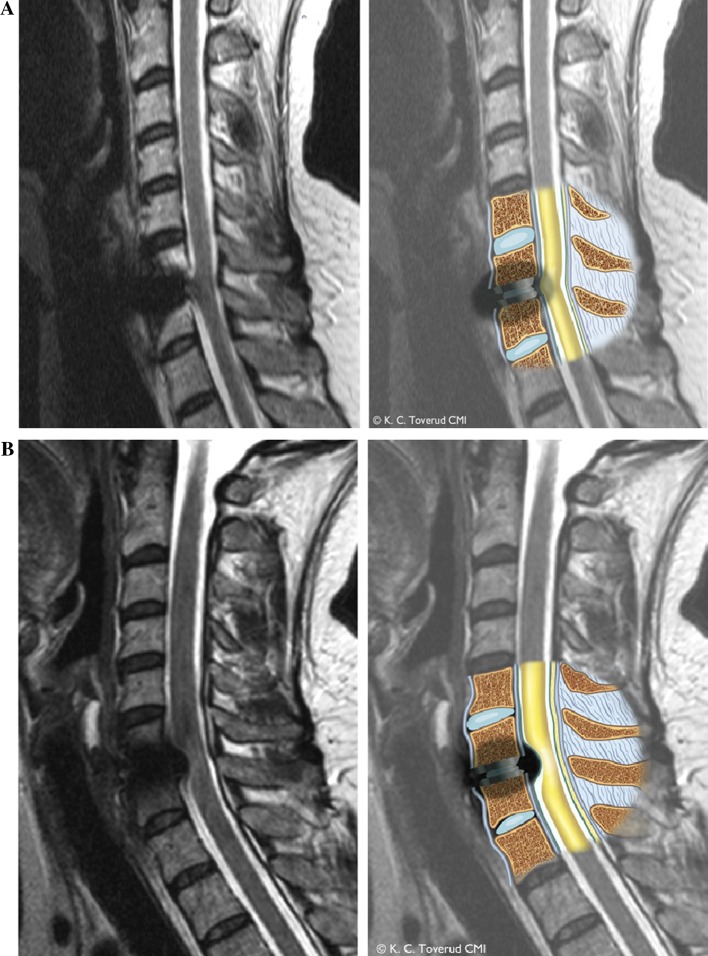
**a** *Left* 1.5 T MRI with T2 sagittal images. *Right* 1.5 T MRI with T2 sagittal images and with an artists illustration of the artifact around the disc prosthesis and effect on the spinal cord (illustrated in *yellow*). The artifact gives the impression that the spinal cord is pulled in the anterior direction towards the disc space/prosthesis and with a change in configuration. **b** *Left* 3 T with T2 sagittal images. *Right* 3 T with T2 sagittal images and with an artists illustration of the artifact around the disc prosthesis and effect on the spinal cord (illustrated in *yellow*) The artifact gives the impression that the spinal cord is dislocated in the posterior direction and with a signal change within the cord. The artifact can be misinterpreted as spinal stenosis

In both the 1.5 and 3 T magnets, the sequences conducted were T1 and T2 sagittal and T2 oblique and axial images. Relevant imaging parameters for 3 T were as follows: Turbo-Spin-Echo (TSE); 3 mm slice thickness; 618/7.8 (repetition time msec/echo time msec) for sagittal T1, 3196/100 for sagittal T2, 4057/100 for oblique T2 and 4109/100 for axial T2; matrix size 312 × 312 for sagittal T1 and for sagittal and oblique T2 and 188 × 187 for axial T2; bandwidth 410.9 Hz/px for T1, 406.4 for sagittal T2, 434.8 for oblique T2 and 404.7 for axial T2;. Relevant imaging parameters for 1.5 T were as follows: TSE; 3 mm slice thickness 552/13 (repetition time msec/echo time msec) for sagittal T1 and for sagittal and oblique T2 4500/97, 4 mm axial MEDIC (me2d) with 891/27, 3 mm axial T2 4000/119; matrix size 512 × 384 for sagittal T1, matrix size 256 × 512 for sagittal and oblique T2, matrix size 256 × 256 for axial T2, matrix size 256 × 192 interpolated to 512 for MEDIC (me2d); bandwidth 130 Hz/px for sagittal and oblique T2, 150 for sagittal T1, 190 for axial T2 and 195 for Medic (me2d). The sagittal T1 and T2 sequences were performed in order to evaluate to what extent artifacts at the operated level impede the interpretation of images in relation to the spinal cord (Fig. 1a, b; Table 1). The oblique and axial images were primarily used to evaluate the root canals (Fig. 3a, b).

**Table 1:** Disc levels possible to assess with 1.5 and 3 T MRI

Evaluated level	Sagittal T2	Axial Medic 2	Axial T2^a^	Sagittal T2	Axial T2	Oblique T2	Oblique T2
	1.5 T	1.5 T	1. 5 T	3 T	3 T	1.5 T	3 T
*N*	10	10	1	10	10	10	10
Adjacent upper level	10	10	1	10	10	10	10
Operated level spinal cord	0	0	0	0	0	–	–
Disc	0	0	0	0	0	–	–
Right foramen	–	0	1	–	1	1	0
Left foramen	–	0	1	–	2	3	1
Adjacent lower level	10	10	1	10	10	10	10

The number indicating how many adjacent upper and lower levels and operated level spinal cord, disc, right and left foramina that was possible to evaluate with the different imaging sequences

*N* number of patients, – not evaluated

^a^ T2 axial 1.5 T was only performed in one patient and the root canals were evaluable at operated level in this patient

**Fig. 2:**
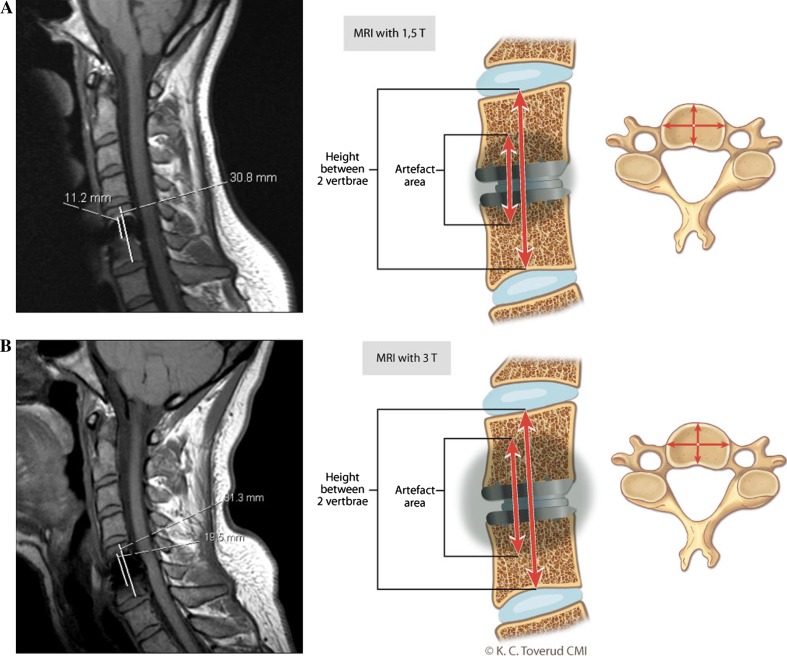
*Left* **a** and **b** T1 sagittal images (**a** 1.5 T and **b** 3 T) with the hight of the artifact and the hight between the upper and lower endplate for the two adjacent vertebrae shown with *lines*. *Right* **a** and **b** the artists illustration of the amount of artifact produced around the titanium prostheses in **a** 1.5 and **b** 3 T MRI. The artifact ratios were calculated by measuring the hight of the artifact cranial and caudal to the prostheses measured at the center of the vertebrae divided with the hight between the upper and lower endplate for the two adjacent vertebrae

The images were evaluated twice by two experienced neuroradiologists and evaluated by consensus. Emphasis were made on signal changes in medulla at all levels, operated level-spinal canal, operated level-disc, operated level-right foramen, operated level-left foramen and on the adjacent upper and lower level-spinal canal, adjacent upper and lower level-disc, adjacent upper and lower level-right foramen, adjacent upper and lower level-left foramen with respect to operated level. The radiologists were blinded with respect to the clinical outcome. A “blur artifact ratio” was calculated and defined as the height of the blur artifact on T1 sagittal images, measuring the “blur” height at midline, from cranial to caudal end of the artifact with respect to height at midline from the superior to inferior endplate of the vertebrae (Fig. 2).

The study was approved by Regional Committees for Medical and Health Research Ethics and by the Data Protection Official for Research.

## Results

**Fig. 3:**
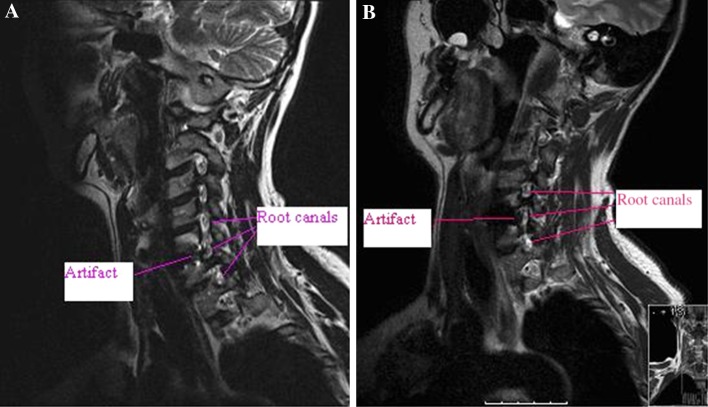
**a** 1.5 T oblique view showing the artifact at operated level C5/C6 and adjacent upper and lower-level root canals. **b** 3 T oblique view showing the artifact at operated level C5/C6 and adjacent upper and lower-level root canals

The superior and inferior adjacent levels with spinal cord and root canals were completely visualized in all patients in both 1.5 and 3 T magnets. However, at the operated level, it was not possible to evaluate the spinal cord and hardly possible to assess the root canals in either of the magnets due to artifacts. The type of artifacts at the operated level was different in the two magnets. With a standard sagittal T2 sequence, the spinal cord seems drawn anteriorly towards the disc space/prostheses in a 1.5 T magnet (Fig. 1a). In the 3 T sagittal T2 sequence, the spinal cord on the other hand seems compressed from the artifact giving the impression of a spinal stenosis with signal change inside the cord (Fig. 1b). In both magnets the image quality is deteriorated to such an extent that interpretation of the spinal cord is impossible in all 10 investigations. With respect to the evaluation of the root canals, the oblique T2 sagittal images at operated level in the 1.5 T magnet was only possible to assess in one right root canal and three left root canals. In the 3 T MR only one left root canal could be evaluated (Fig. 3a, b).

The axial images in the 1.5 T magnet were assessed using the MEDIC sequences as part of a standard protocol which is known to reduce the signal changes produced by CSF flow around the spinal cord. There were substantial artifacts present at the operated level despite minimized disturbances from CSF flow, making it impossible to evaluate the root canals adequately. In one patient an axial T2 sequence was done in addition to the MEDIC in the 1.5 T magnet, which improved visualization of the root canals at operated level. In the 3 T magnet the T2 axial sequences were used in all 10 patients. It was only possible to evaluate both root canals in one patient and one root canal in another. In the rest, the root canals were impossible to evaluate.

The mean “blur artifact ratio” was 47.0 % (range 39.2–58.2) in the 1.5 T magnet and 54.2 % (range 30.1–71.4) in the 3 T magnet, (Students *t* test, *p* = 0.132).

To make sure none of the patients had persistent root canal stenosis misinterpreted as artifact on MRI, we controlled the clinical outcome at one year and found that there was one patient who experienced persistent symptoms in his left arm after surgery. In this patient the root canals were visualized and evaluated as open in both 1.5 and 3 T magnets.

The endomedullary high signal intensity observed at operated level was attributed to artifact from implant, due to lack of corresponding clinical signs of myelopathy.

## Discussion

The present study shows that the artifact caused by the Discover^®^ titanium cervical disc prosthesis makes the images difficult to interpret at operated level with respect to the spinal cord and root canals in both 1.5 and 3 T cervical MRI,. The adjacent levels were, however, well visualized in both magnets.

Metal cause artifacts on MRI, the extent being dependent of many factors, among them the alloy composition of the implant. A ferromagnetic alloy such as Iron, Nickel and Cobalt produce more extensive artifacts than non-ferromagnetic materials such as Titanium. Sekhon et al. [18] found that the image quality with the ferromagnetic cobalt-chrome metal alloys in the Prodisc-C^®^ (Synthes Spine, Paoli PA) and the PCM^®^ (Cervitech, Rockaway, NJ) prostheses where significantly deteriorated compared to the titanium produced Bryan^®^ disc (Medtronic Sofamore Danek, Memphis TN) and Prestige LP^®^ (Medtronic Sofamore Danek) prostheses. Titanium induces fewer artifacts than the ferromagnetic materials and is recommended as implant material in a patient who may need further MR examination [14]. The prostheses used in our study, is made of titanium and could thus be an alternative when postoperative MRI assessments are needed. However, artifacts were found at operated level in all patients, making a diagnostic evaluation of operated level highly restricted.

Higher magnet field strength produces a greater degree of artifact with an increased artifact ratio in 3 T compared to 1.5 T magnets. The same has been reported in a recent study which compared the image quality in a 1.5 T magnet to the image quality in an open 0.2 T unit after implantation of a cobalt-chrome-molybdenum alloy (Co-Cr) cervical disc arthroplasty. The 0.2 T MRI reduced the magnitude of artifact without a significant reduction in image quality [19]. Gerigk et al. [20] found that when a Titanium cage is used, 1.5 T magnet facilitates multiplanar and transforaminal reconstructions, and allows foraminal narrowing to be assessed. Sekhon et al. also found that in a 1.5 T magnet with T2 axial images, visualization of neural structures at both operated and adjacent levels were possible in the presence of a titanium implant. This was not confirmed in the present study, where an adequate interpretation of the spinal cord and root canals at operated level were not possible in either 1.5 or 3 T MRI due to the artifacts induced. These results are also in contrast to a previous study that described satisfactory visualization of the spinal cord at operated level with a titanium cervical disc arthroplasty in a 1.5 T magnet on T2 weighted images [18]. Reduced visualization of root canals at operated level, may be explained by different sequence parametres conducted in 1.5 T in our study versus previously reported by others. In particular, will the axial MEDIC sequence give artifact in presence of metal. This sequence is performed for good evaluation of disc protrusion and CSF space evaluation avoiding CSF flow disturbance. The axial MEDIC sequence in the Siemens 1.5 T magnet, weighting from the combination of several gradient echoes produces a much higher signal to noise ratio (SNR) [21], but was in our study without value in the presence of the Discover^®^ titanium disc prosthesis.

However, evaluation of the spinal cord with sagittal images was performed with standard T2 weighted sequences in all studies. Various findings may thus not be explained by the image sequences selected. Different disc prostheses with differing amount of titanium may be another explanation for the variety of findings. As regard to 3 T MRI, we could not find comparable studies in the literature.

Adjusting the MR sequences to produce the least amount of artifact and to select the optimal image sequences is important. Spin-echo sequences reduce the size of the artifact. In addition frequency encoding direction, slice thickness, bandwidth and echo-time will influence the extent of artifacts. In our study these factors were not investigated. In the future there is expected to be novel MR techniques for metal artifact reduction available from most vendors for both 1.5 and 3 T magnets. Meanwhile alternative imaging modalities may be necessary in evaluation of operated patients with persistent clinical symptoms.

The axial and oblique T2 sequences were significantly impeded in both magnets. The T2 weighted images of the spinal cord at the index level were also impossible to interpret in both 1.5 and 3 T magnets. The spinal cord on the sagittal T2 images seemed drawn anteriorly towards the disc space/prostheses in the 1.5 T field strength, while compressed in the 3 T with the impression of a spinal stenosis and high signal change within the cord.

The introduction of artificial cervical discs has provided a great challenge in post-operative radiological diagnosis. Robinson and Sanden [22] concluded that in the presence of a steel alloy lumbar artificial disc, alternative imaging modalities should be evaluated. In the present study, where a titanium implant was used, our conclusion is the same when the objective is to visualize the spinal cord and root canals at operated level.

Regarding PEEK cages used as cervical disc implant in ACDF, the artifacts induced in MRI are reported to be minimal, making both the spinal cord and root canals well visualized [23]. Although there is no strong evidence to support the routine use of ACDA over ACDF in single-level cervical spondylosis [9, 10, 24], ACDA is still widely used as an alternative to fusion where the main purpose is to preserve motion after discectomy.

### Limitations of our study

The lack of significance when comparing 1.5 versus 3 T images and “blur artifact ratios”, might be explained by the low power of the study due to the small sample size.

The study is a small pilot study focusing on one specific titanium implant.

The imaging parameters of the 1.5 and 3T sequences were not similarly optimized in relation to the metal implant.

Different axial sequences in the two magnets make it impossible to compare evaluation of the root canals using this sequence.

## Conclusion

The Discover^®^ titanium cervical disc prostheses induce significant artifact in both 1.5 and 3 T MRI and most pronounced in 3 T. The artifacts were of different character in the two magnets. Evaluation of the spinal cord at the operated level was not possible in either the 1.5 T or the 3 T examination. Visualization of the root canals at the operated level is difficult with both magnet strengths. Concerns regarding future need of visualization of the operated level arise if ACDA is to be used and surgeons should be aware of the difficulty in evaluation of operated disc level with MRI. Novel MR techniques for metal artifact reduction should be evaluated. Meanwhile, alternative imaging modalities may be needed for follow-up assessment of the operated level. 
